# Usher Syndrome Type 2D Associated With a Novel Homozygous Whrn c.74dup Variant: A Case Report

**DOI:** 10.7759/cureus.110478

**Published:** 2026-06-08

**Authors:** Emilio A Cepeda Terrasa, Adriana Ramirez, Natalio Izquierdo

**Affiliations:** 1 Department of Ophthalmology, School of Medicine, Medical Sciences Campus, University of Puerto Rico, San Juan, PRI; 2 Department of Ophthalmology, Ponce Health Sciences University, Ponce, PRI

**Keywords:** case report, eog, erg, genetic testing, inherited retinal disease, oct, retinitis pigmentosa, rod-cone dystrophy, usher syndrome, whrn

## Abstract

Variants of the WHRN/DFNB31 gene cause Usher syndrome type 2D (USH2D), a rare subtype of Usher syndrome. Usher syndrome is an autosomal recessive disorder characterized by sensorineural hearing loss and progressive retinal degeneration due to retinitis pigmentosa. We report the case of a 63-year-old woman with clinical and genetic findings consistent with USH2D. Ophthalmic evaluation demonstrated reduced visual acuity, bilateral optic disc pallor, mid-peripheral bony spicules, vascular attenuation, and macular abnormalities. Full-field electroretinography showed non-recordable photopic and scotopic responses bilaterally, consistent with extinguished rod and cone responses. Macular optical coherence tomography demonstrated a central subfield thickness of 220 µm (oculus dexter or right eye (OD)) and 235 µm (oculus sinister or left eye (OS)), a cube volume of 7.8 mm³ OD and 6.5 mm³ OS, and an average cube thickness of 215 µm OD and 180 µm OS, supporting structural macular involvement. Electrooculography demonstrated an abnormal Arden ratio of 1.58 in both eyes, consistent with retinal pigment epithelium dysfunction. Based on these ocular findings, the patient was diagnosed with retinitis pigmentosa. Genetic testing with next-generation sequencing identified a homozygous pathogenic WHRN c.74dup (p.Gly26Argfs*153) variant. To our knowledge, this specific frameshift variant has not been previously reported in the literature in affected individuals with WHRN-related disease. This case highlights the importance of genetic testing and multimodal ophthalmic evaluation in patients with inherited retinal dystrophies and contributes to the limited literature describing Usher syndrome due to WHRN mutations.

## Introduction

Usher syndrome is the most common inherited cause of combined sensorineural hearing loss and progressive visual impairment due to retinitis pigmentosa [1]. This inherited disease is clinically and genetically heterogeneous and is classified into three subtypes based on the severity of hearing loss, vestibular dysfunction, and onset of visual symptoms [1].

Usher syndrome type 1 (USH1) is the most severe subtype and is characterized by profound congenital sensorineural hearing loss, early-onset retinitis pigmentosa, and vestibular dysfunction leading to delayed motor development [1]. Usher syndrome type 2 (USH2) is the most common subtype and is generally characterized by moderate-to-severe congenital hearing loss, progressive retinal degeneration, and preserved vestibular function [2]. Usher syndrome type 3 (USH3) is less common and is characterized by progressive hearing loss during adulthood, variable vestibular dysfunction, and later-onset retinitis pigmentosa [1,2]. Ophthalmic manifestations in patients with Usher syndrome frequently include nyctalopia, progressive peripheral visual field loss, and retinal pigment epithelium changes associated with retinitis pigmentosa [1,2]. Together, the pattern of auditory involvement, vestibular findings, and retinal degeneration guides the clinical diagnosis and classification of Usher syndrome [1,2].

Usher syndrome affects approximately 4-17 individuals per 100,000 worldwide and exhibits variable clinical expressivity, which may complicate diagnosis and management [1,2].

The genetic basis of Usher syndrome involves mutations in several genes responsible for maintaining the structure and function of inner ear hair cells and retinal photoreceptors [2,3]. Mutations in WHRN/DFNB31 have been associated with Usher syndrome type 2D (USH2D), a rare subtype within the broader USH2 classification [3,4]. Mutations in this gene were originally implicated in USH2D after Ebermann and coworkers identified mutations in the long isoform of whirlin in patients with retinitis pigmentosa and sensorineural hearing loss [4].

The WHRN gene encodes whirlin, a scaffold protein expressed in multiple isoforms [5,6]. Whirlin plays an important role in the organization and elongation of stereocilia in cochlear hair cells and in maintaining the structural integrity of retinal photoreceptors [7,8]. Alterations in this protein may lead to dysfunction of sensory cells responsible for hearing and vision, contributing to the phenotype observed in affected patients [9].

To our knowledge, reports exploring mutations in WHRN leading to Usher syndrome remain limited in the current literature [10]. For this reason, further phenotypic characterization of these cases is important to better understand the clinical manifestations associated with WHRN variation [3].

We report the case of a 63-year-old woman with Usher syndrome associated with a homozygous pathogenic WHRN variant who had late-stage rod-cone dystrophy. This case contributes to the limited literature on these genetic variants and provides further information on the phenotype of patients with WHRN mutations leading to Usher syndrome.

## Case presentation

A 63-year-old woman with a past medical history of rheumatoid arthritis, hypertension, hypercholesterolemia, and hypothyroidism was referred to our clinic for genetic evaluation. Family history was unremarkable, and the patient denied consanguinity.

Upon initial comprehensive ophthalmic evaluation by at least one of the authors (NJI), the patient had a best corrected visual acuity of 20/200 in the right eye (oculus dexter or right eye (OD)) and 20/60 in the left eye (oculus sinister or left eye (OS)). Intraocular pressure was 16 mmHg OD and 15 mmHg OS. She had bilateral posterior chamber intraocular lenses; however, prior cataract surgery records were not available for review.

Indirect ophthalmoscopy showed waxy optic nerve pallor with peripapillary atrophy, macular degeneration, and peripheral bone spicules. Full-field electroretinography (ERG) demonstrated non-recordable scotopic and photopic responses in both eyes. Flicker responses were diminished, latencies were delayed, and oscillatory potentials showed diminished amplitudes. These findings were consistent with severe rod and cone dysfunction.

**Figure 1:**
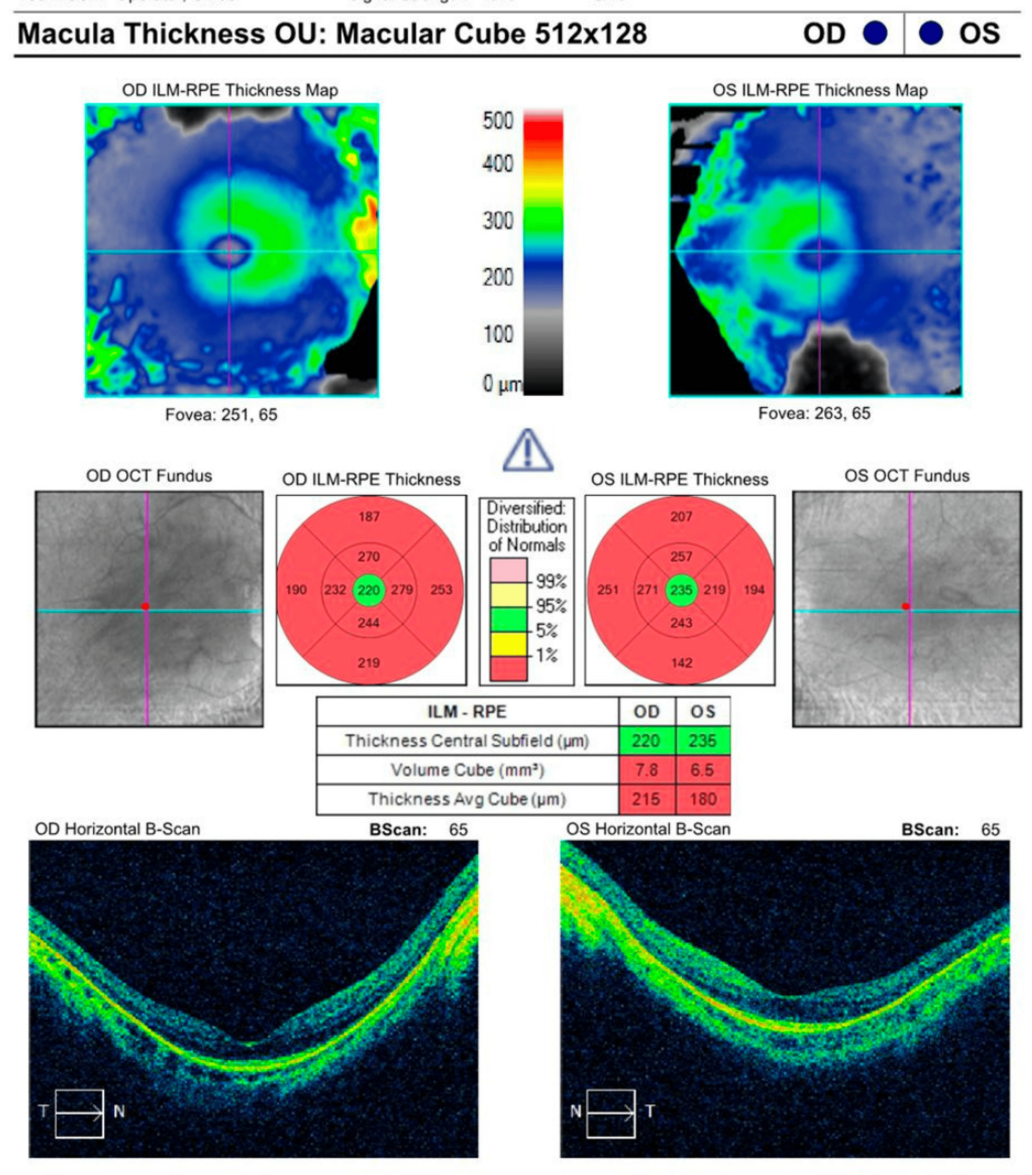
Macular optical coherence tomography study showing decreased macular thickness and volume bilaterally. OCT: Optical coherence tomography; RPE: retinal pigment epithelium; ILM: inner limiting membrane; OD: oculus dexter or right eye; OS: oculus sinister or left eye.

Macular optical coherence tomography (OCT) demonstrated a central subfield thickness of 220 µm OD and 235 µm OS, a cube volume of 7.8 mm³ OD and 6.5 mm³ OS, and an average cube thickness of 215 µm OD and 180 µm OS. These findings suggest structural macular involvement and are consistent with advanced retinal degeneration (Figure 1).

Electrooculography (EOG) demonstrated an abnormal Arden ratio of 1.58 in both eyes below the normal threshold used for clinical interpretation. This abnormal result is consistent with retinal pigment epithelium dysfunction.

An Invitae next-generation sequencing diagnostic test (Laboratory for Molecular Medicine, Center for Genetics and Genomics, Cambridge, Massachusetts) evaluating more than 300 genes associated with inherited retinal diseases was performed. Results showed a homozygous WHRN c.74dup (p.Gly26Argfs*153) frameshift variant, which was classified as pathogenic by the diagnostic laboratory. The variant is predicted to create a premature translational stop signal resulting in an absent or disrupted protein product. It is absent from gnomAD and has not been previously reported in the literature in affected individuals with WHRN-related conditions.

Audiometric data and formal ENT evaluation were not available for review at the time of manuscript preparation. The patient was referred for audiology consultation and was prescribed lutein 20 mg/zeaxanthin 4 mg daily capsules.

## Discussion

Usher syndrome was described by British ophthalmologist Dr. Charles Usher in the early 20th century, when he identified the hereditary association between hearing loss and retinitis pigmentosa [1,11]. The syndrome is characterized by sensorineural hearing loss, progressive retinal degeneration, and variable vestibular dysfunction.

Previous studies have reported that patients with USH2D due to WHRN mutations typically have progressive retinal degeneration consistent with retinitis pigmentosa, supporting the role of WHRN in maintaining retinal photoreceptor structure [3,7]. Our patient had late-stage rod-cone dystrophy characterized by reduced visual acuity, bilateral optic disc pallor, mid-peripheral bone spicules, and macular structural abnormalities on OCT, including decreased macular cube volume and average thickness. The patient's phenotype is compatible with the retinal findings described in patients with Usher syndrome.

Studies in European populations, including Spain, have demonstrated the genetic heterogeneity of Usher syndrome and emphasized the importance of population-specific studies [12,13]. Sequence variants in WHRN/DFNB31 appear to represent a limited proportion of Usher syndrome cases [10]. In our patient, next-generation sequencing identified a homozygous pathogenic WHRN c.74dup (p.Gly26Argfs*153) variant. This finding supports the phenotypic and genotypic diagnosis of WHRN-associated Usher syndrome. To our knowledge, based on the diagnostic genetic report and available published literature, this specific early frameshift variant has not been previously reported in affected individuals with WHRN-related disease. Given Puerto Rico's historical Spanish ancestry, genetic studies from Spanish populations may provide relevant population context for variants identified in Puerto Rican patients; however, a direct relationship with this specific variant cannot be established.

Previous studies have reported that WHRN encodes whirlin, which plays a critical role in the organization of stereocilia in cochlear hair cells and in the structural integrity of retinal photoreceptors [3,14]. Disruption of this protein has been associated with dysfunction of sensory cells responsible for both hearing and vision [5,7]. Notably, the patient's frameshift variant, c.74dup, occurs early in the coding sequence and is predicted to affect the N-terminal region specific to the long whirlin isoform [5,7].

Studies by Mathur, Yang, and coworkers demonstrated that N-terminal mutations disrupt full-length whirlin in both the inner ear and retina, leading to retinal degeneration and moderate-to-severe hearing loss characteristic of USH2D, whereas C-terminal mutations may spare the retina and result in non-syndromic deafness [5,7]. This isoform-specific mechanism may explain the clinical manifestations observed in our patient, including progressive retinal degeneration.

This case addresses an important gap in the literature by contributing to the limited number of reports describing patients with USH2D due to mutations in WHRN. Since patients with Usher syndrome have combined vision and hearing involvement, multidisciplinary care is essential [15].

Limitations of this study include its reliance on a single patient, which limits the ability to generalize these findings to a broader population of patients with Usher syndrome. An additional limitation is the absence of objective audiometric data and formal ENT evaluation, which limits complete characterization of the auditory phenotype. Nevertheless, the diagnosis of USH2D is supported by the homozygous pathogenic WHRN c.74dup (p.Gly26Argfs*153) variant and the patient's advanced retinal phenotype. This highlights the importance of continued characterization of genotype-phenotype relationships, particularly for rare variants, to improve patient counseling, prognostication, and the future development and application of emerging genetic therapies in Usher syndrome [6].

Studies including larger patient populations specifically evaluating WHRN mutations in Usher syndrome are warranted. Further research should aim to investigate the underlying mechanisms and clinical variability of this condition.

## Conclusions

This case describes a rare presentation of USH2D associated with a homozygous pathogenic WHRN c.74dup (p.Gly26Argfs*153) variant. The patient's multimodal ophthalmic findings, including fundus examination, OCT, ERG, and EOG, were consistent with advanced rod-cone dystrophy. This report contributes to the limited literature describing WHRN-associated Usher syndrome and highlights the importance of genetic testing, multimodal ophthalmic evaluation, and multidisciplinary care in patients with inherited retinal dystrophies.
